# DNA methylation-based biological age, genome-wide average DNA methylation, and conventional breast cancer risk factors

**DOI:** 10.1038/s41598-019-51475-4

**Published:** 2019-10-21

**Authors:** Minyuan Chen, Ee Ming Wong, Tuong L. Nguyen, Gillian S. Dite, Jennifer Stone, Pierre-Antoine Dugué, Graham G. Giles, Melissa C. Southey, Roger L. Milne, John L. Hopper, Shuai Li

**Affiliations:** 1 Centre for Epidemiology and Biostatistics, Melbourne School of Population and Global Health, University of Melbourne, Melbourne, Australia; 2 International Federation of Red Cross and Red Crescent Societies Country Cluster Support Team, Beijing, China; 3 Genetic Epidemiology Laboratory, Department of Clinical Pathology, University of Melbourne, Melbourne, Australia; 4 Precision Medicine, School of Clinical Sciences at Monash Health, Monash University, Monash, Australia; 5 Centre for Genetic Origins of Health and Disease, Curtin University and the University of Western Australia, Perth, Australia; 6 Cancer Epidemiology Division, Cancer Council Victoria, Melbourne, Australia; 7 Centre for Cancer Genetic Epidemiology, Department of Public Health and Primary Care, University of Cambridge, Cambridge, UK

**Keywords:** Breast cancer, DNA methylation, Risk factors

## Abstract

DNA methylation-based biological age (DNAm age), as well as genome-wide average DNA methylation, have been reported to predict breast cancer risk. We aimed to investigate the associations between these DNA methylation-based risk factors and 18 conventional breast cancer risk factors for disease-free women. A sample of 479 individuals from the Australian Mammographic Density Twins and Sisters was used for discovery, a sample of 3354 individuals from the Melbourne Collaborative Cohort Study was used for replication, and meta-analyses pooling results from the two studies were conducted. DNAm age based on three epigenetic clocks (Hannum, Horvath and Levine) and genome-wide average DNA methylation were calculated using the HumanMethylation 450 K BeadChip assay data. The DNAm age measures were positively associated with body mass index (BMI), smoking, alcohol drinking and age at menarche (all nominal *P* < 0.05). Genome-wide average DNA methylation was negatively associated with smoking and number of live births, and positively associated with age at first live birth (all nominal *P* < 0.05). The association of DNAm age with BMI was also evident in within-twin-pair analyses that control for familial factors. This study suggests that some lifestyle and hormonal risk factors are associated with these DNA methylation-based breast cancer risk factors, and the observed associations are unlikely to be due to familial confounding but are likely causal. DNA methylation-based risk factors could interplay with conventional risk factors in modifying breast cancer risk.

## Introduction

DNA methylation has been proposed to play an important role in the aetiology of complex traits and diseases, including cancer^1,2^. DNA methylation, especially when detected in blood, has been investigated for its association with breast cancer risk. Peripheral blood DNA methylation at the known breast cancer susceptibility genes *BRCA1* and *ATM* has been found to be associated with elevated breast cancer risk^3–5^. Several genome-wide studies of DNA methylation have reported peripheral blood DNA methylation changes associated with the risk of breast cancer^6–10^.

Two DNA methylation-based measures have been reported to be associated with breast cancer risk. One measure is DNA methylation-based biological age (DNAm age). Several epigenetic clocks have been developed to estimate DNAm age^11^, and the Hannum^12^ and Horvath^13^ clocks have received the most attention. Both these clocks are developed by regressing chronological age on individual methylation sites to select a set of sites to predict chronological age. The Levine clock^14^ has recently been developed by selecting a set of sites to predict ‘phenotypic age’, a linear combination of 10 clinical biomarkers (including chronological age) associated with the hazard of aging-related mortality. The difference between DNAm age and chronological age is called ‘epigenetic age acceleration’, which reflects the rate of biological aging. A positive value of epigenetic age acceleration suggests that biological age is older than chronological age. Prospective analyses have found associations between accelerated DNAm ages based on the Horvath and Levine clocks and increased risk of breast cancer for middle-aged women^14,15^. Recently, a prospective study assessed DNAm age for 2,764 middle-aged women using DNA extracted from pre-diagnosis blood samples and found that each of the three clocks above was predictive of breast cancer risk: each 5-year increase in epigenetic age acceleration (calculated as the residuals from regressing DNAm age on chronological age and blood cell composition and thus independent of chronological age and blood cell composition) was associated with about a 10–15% increase in breast cancer risk^16^.

Another DNA methylation measure reported to be associated with breast cancer risk is a global measure, genome-wide average DNA methylation, defined as the average methylation value across multiple probes, commonly calculated from the HumanMethylation 450 K (HM450) BeadChip^17,18^. Two studies found that a lower genome-wide average DNA methylation in pre-diagnosis peripheral blood was associated with increased risk of breast cancer^8,9^. These results are consistent with previous findings that conventional global blood DNA methylation measures, such as LINE-1 and LUMA, are negatively associated with breast cancer risk^19,20^. However, this association was not observed in the Norwegian Women and Cancer Study^9^, the Sister Study^10^ or a recent meta-analysis of four studies^21^, two of which are the studies of Refs. 8 and 9. A similar measure, the median methylation value across probes, has been found to be prospectively associated with risks of mature B-cell neoplasms^22^, urothelial cell carcinoma^23^ and prostate cancer^24^.

These measures could be treated as putative DNA methylation-based breast cancer risk factors. It is unknown whether these DNA-methylation based risk factors and conventional risk factors modify breast cancer risk independently or in combination. Knowing the associations between the two groups of risk factors could be helpful for answering this question and provide insights into breast cancer aetiology and risk prediction. As Christensen pointed out^25^: “in-depth studies of both established and putative breast cancer risk factors for their relationship with epigenetic age acceleration can add to our understanding of the biology underlying disease risk factors and present new opportunities for primary and secondary prevention of breast cancer.”

DNAm age has been investigated for its associations with lifestyle factors and some inconsistent results were reported^11^. Levine *et al*.^26^ found age at menopause was negatively associated with accelerated DNAm age based on the Horvath clock, opposite to the direction of the two factors’ associations with breast cancer risk. From a twin and family study, we found evidence that the variance in genome-wide average DNA methylation is determined by (unmeasured) environmental factors across the lifespan^17^; no specific environmental factor was investigated. To the best of our knowledge, associations of these DNA methylation-based risk factors with conventional breast cancer risk factors, such as family history or mammographic density, have not been investigated.

We therefore conducted a study with the aim of investigating associations between DNA methylation-based risk factors and conventional risk factors for breast cancer.

## Results

### Characteristics of the Australian mammographic density twins and sisters study (AMDTSS) sample

Table 1 shows the distribution of the investigated risk factors for the AMDTSS sample. Our sample is comparable with samples from previous studies^8,9,14,15^ that reported on the DNA methylation-based breast cancer risk factors considered in this study.

**Table 1:** Characteristics of the study sample.

Characteristics	Monozygotic twins	Dizygotic twins	Sisters
Approximately normal variables, mean (standard deviation)			
Age (years)	55.6 (8.4)	57 (7.2)	56.6 (8.0)
DNAm age (years)			
Hannum clock	56.3 (6.3)	58.4 (5.9)	57.2 (6.6)
Horvath clock	54.8 (6.7)	56.5 (6.0)	55.5 (6.5)
Levine clock	52.1 (6.9)	53.6 (7.2)	53.2 (7.7)
Epigenetic age acceleration (years)			
Hannum clock	−0.5 (3.9)	0.7 (4.2)	−0.1 (4.4)
Horvath clock	−0.4 (4.4)	0.6 (4.8)	−0.1 (4.9)
Levine clock	−0.4 (4.8)	0.2 (5.9)	0.1 (5.8)
Genome-wide average DNA methylation (percentage)	53.0 (0.3)	53.0 (0.3)	53.0 (0.3)
Age at menarche (years)	13.0 (1.3)	12.9 (1.5)	13.0 (1.5)
Age at menopause (years)^a^	47.0 (5.9)	45.8 (6.3)	45.8 (6.0)
Age at first live birth (years)^b^	25.1 (5.3)	25.0 (4.7)	24.7 (4.8)
Skewed variables, median (inter-quartile range)			
Body mass index	24.7 (22.7–30.0)	26.4 (23.0–29.1)	25.7 (23.0–30.1)
Pack-years of smoking^c^	9.0 (3.6–21.2)	7.0 (2.0–14.3)	5.0 (1.9–13.4)
Number of live births^b^	3.0 (2.0–3.3)	3.0 (2.0–4.0)	3.0 (2.0–4.0)
Years of hormonal replacement therapy use^d^	2.0 (0.8–6.3)	4.8 (1.3–10.8)	3.4 (1.2–7.0)
Years of oral contraceptive use^e^	5.0 (2.0–9.5)	5.0 (2.0–10.0)	5.0 (1.8–11.7)
Mammographic density^f^			
Dense area	26.4 (6.6–46.7)	26.5 (7.9–46.3)	25.7 (12.4–44.9)
Non-dense area	109.0 (66.8–142.2)	94.7 (63.0–136.5)	98.0 (68.6–148.5)
Percentage dense area	19.4 (4.2–41.8)	23.0 (7.3–39.8)	21.5 (9.9–37.7)
Binary variables, N (%)			
Smoking	49 (37.1%)	47 (35.6%)	92 (42.8%)
Alcohol drinking	77 (58.3%)	80 (60.6%)	130 (60.5%)
Post-menopausal	81 (61.4%)	102 (77.3%)	145 (67.4%)
Parous	116 (87.9%)	117 (88.6%)	198 (92.1%)
Hormonal replacement therapy use	43 (32.6%)	58 (43.9%)	85 (39.5%)
Oral contraceptive use	106 (80.3%)	111 (84.1%)	183 (85.1%)
Family history	32 (24.2%)	14 (10.6%)	45 (20.9%)

^a^For post-menopausal women; ^b^For parous women; ^c^For ever smokers; ^d^For hormonal replacement therapy users; ^e^For oral contraceptive users; ^f^Sample sizes were 122, 123 and 190 for monozygotic twins, dizygotic twins and sisters, respectively.

The mean (SD) DNAm age based on the Hannum, Horvath and Levine clocks were 57.3 (6.4), 55.5 (6.5) and 53.0 (7.4) years, respectively. By construction, the mean epigenetic age acceleration was zero years, and the SD was 4.2, 4.7 and 5.6 years, respectively. The mean (SD) percentage genome-wide average DNA methylation was 53.0% (0.3%).

**Figure 1:**
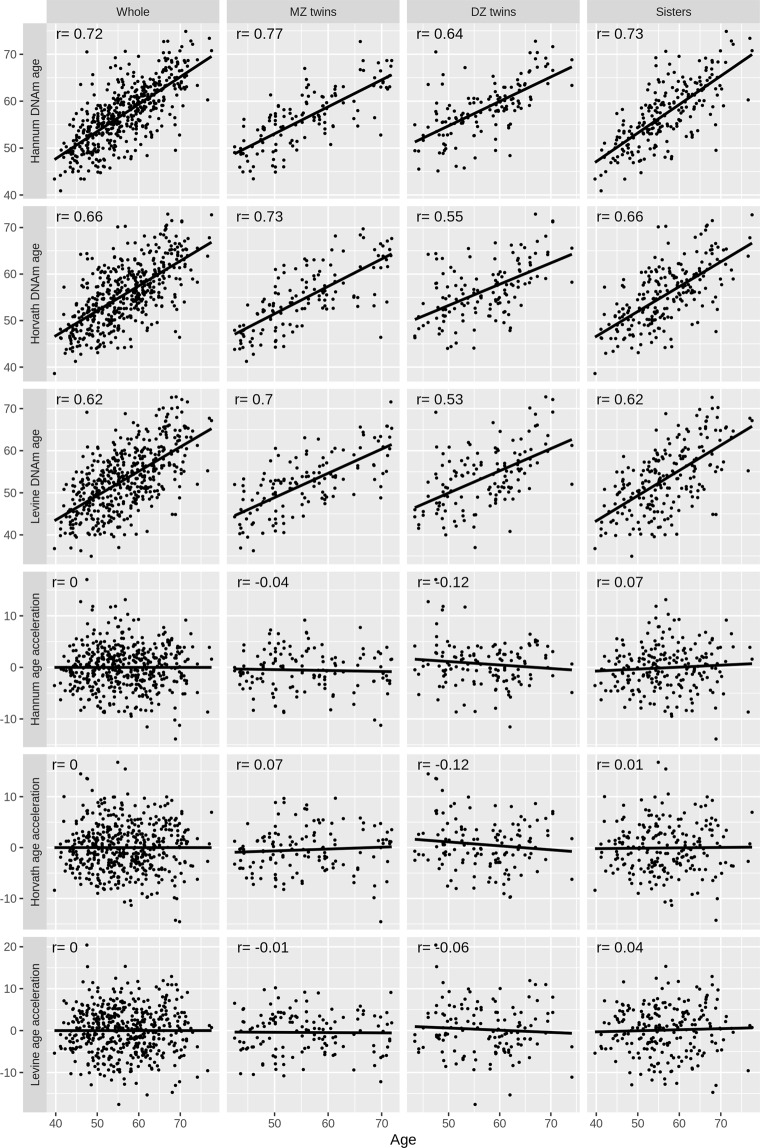
Correlation of chronological age with DNAm age.

**Figure 2:**
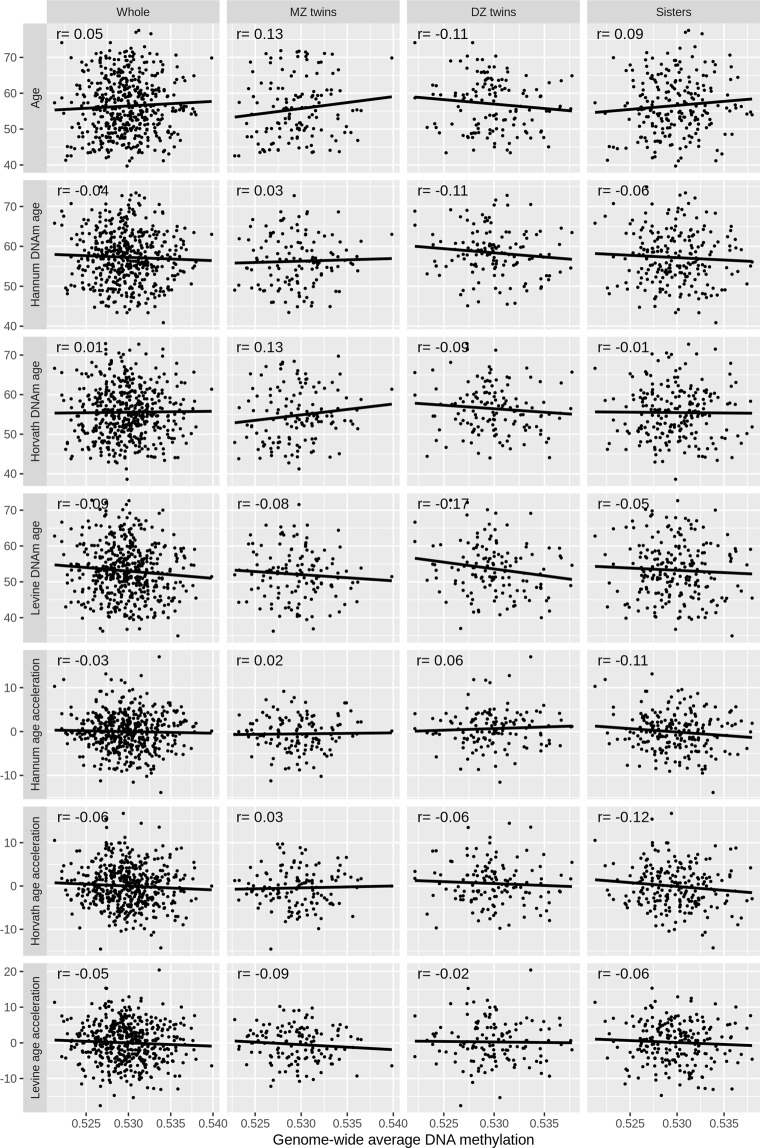
Correlations of genome-wide average DNA methylation with chronological ages and DNAm age.

Figures 1 and 2 show the correlations between chronological age and the DNA methylation-based risk factors. The three DNAm age measures were correlated with each other, and each of them was correlated with chronological age (all r > 0.61; all *P* < 10^−15^). The three epigenetic age acceleration measures were correlated with each other (all r > 0.62; all *P* < 10^−15^), and independent of chronological age by construction. Genome-wide average DNA methylation was independent of chronological age, DNAm age and epigenetic age acceleration (all *P* > 0.19). No obvious heterogeneity in the correlations across MZ twins, DZ twins and sisters was observed.

### Associations between DNA methylation-based and conventional risk factors

Table 2 shows that the epigenetic acceleration measures were associated with different conventional risk factors: the Hannum clock was negatively associated with age at first live birth, the Horvath clock was positively associated with BMI and age at menarche, and the Levine clock was positively associated with BMI, pack-years of smoking and alcohol drinking (all nominal *P* < 0.05); BMI was the only risk factor associated with two epigenetic acceleration measures. Genome-wide average DNA methylation was negatively associated with the number of live births, and positively associated with age at first live birth (both nominal *P* < 0.05). No association was found for the other risk factors.

**Table 2:** Association estimates between epigenetic age acceleration, genome-wide average DNA methylation and conventional breast cancer risk factors

Conventional risk factors	Hannum clock		Horvath clock		Levine clock		Genome-wide average DNA methylation	
	β (95% CI)	*P*	β (95% CI)	*P*	β (95% CI)	*P*	β (95% CI)^a^	*P*
Body mass index (n = 479)	1.51 (−0.72, 3.75)	0.18	2.35 (0.05, 4.66)	0.05	3.12 (0.37, 5.86)	0.03	−0.06 (−0.20, 0.07)	0.38
Ever smoking (n = 479)	−0.41 (−1.21, 0.40)	0.32	0.28 (−0.64, 1.20)	0.55	0.45 (−0.60, 1.51)	0.40	−0.03 (−0.09, 0.02)	0.20
Pack-years of smoking (n = 188)	0.21 (−0.12, 0.54)	0.21	0.12 (−0.35, 0.59)	0.61	0.72 (0.27, 1.16)	0.002	0.003 (−0.03, 0.03)	0.86
Ever alcohol drinking (n = 287)	0.12 (−0.67, 0.91)	0.76	0.81 (−0.17, 1.79)	0.11	1.21 (0.18, 2.25)	0.02	−0.01 (−0.07, 0.04)	0.65
Age at menarche (n = 479)	0.13 (−0.11, 0.38)	0.28	0.31 (0.01, 0.61)	0.04	0.23 (−0.10, 0.57)	0.16	0.01 (−0.01, 0.03)	0.24
Post-menopausal (n = 479)	0.73 (−0.61, 2.07)	0.28	−0.49 (−1.77, 0.79)	0.45	−0.29 (−1.77, 1.20)	0.71	0.06 (−0.02, 0.14)	0.18
Age at menopause (n = 328)	−0.03 (−0.10, 0.05)	0.44	−0.05 (−0.14, 0.04)	0.29	−0.05 (−0.16, 0.07)	0.42	0.001 (−0.01, 0.01)	0.75
Parity (parous; n = 479)	−0.38 (−1.87, 1.10)	0.61	−0.57 (−2.06, 0.92)	0.46	−0.15 (−2.08, 1.79)	0.88	0.04 (−0.04, 0.12)	0.35
Number of live births (n = 431)	0.25 (−0.62, 1.13)	0.57	0.04 (−1.19, 1.27)	0.95	0.15 (−1.03, 1.33)	0.81	−0.08 (−0.14, −0.02)	0.01
Age at first live birth (n = 431)	−0.10 (−0.20, −0.01)	0.03	−0.07 (−0.18, 0.04)	0.18	−0.10 (−0.22, 0.02)	0.11	0.01 (0.001, 0.014)	0.02
Ever HRT use (n = 479)	0.53 (−0.37, 1.44)	0.25	−0.04 (−0.98, 0.89)	0.93	0.20 (−0.93, 1.33)	0.73	0.03 (−0.03, 0.09)	0.27
Years of HRT use (n = 186)	0.34 (−0.11, 0.79)	0.14	0.21 (−0.35, 0.78)	0.46	0.49 (−0.13, 1.10)	0.12	−0.002 (−0.03, 0.03)	0.90
Ever oral contraceptive use (n = 479)	−0.95 (−2.01, 0.10)	0.08	0.17 (−0.95, 1.30)	0.76	−0.48 (−1.83, 0.86)	0.48	−0.03 (−0.09, 0.04)	0.39
Years of oral contraceptive use (n = 400)	−0.19 (−0.52, 0.13)	0.25	−0.05 (−0.43, 0.34)	0.80	−0.09 (−0.51, 0.33)	0.68	−0.02 (−0.04, 0.004)	0.12
Having family history (n = 479)	0.04 (−1.23, 1.31)	0.95	0.36 (−1.02, 1.74)	0.61	0.28 (−1.27, 1.83)	0.72	0.03 (−0.02, 0.09)	0.25
Mammographic dense area (n = 435)	0.14 (−0.76, 1.04)	0.75	−0.01 (−0.99, 0.96)	0.98	0.62 (−0.45, 1.68)	0.26	−0.02 (−0.07, 0.03)	0.38
Mammographic non-dense area (n = 435)	−1.16 (−2.42, 0.10)	0.07	−1.03 (−2.33, 0.27)	0.12	−0.98 (−2.41, 0.45)	0.18	0.06 (−0.03, 0.15)	0.20
Mammographic percentage dense area (n = 435)	0.14 (−0.27, 0.55)	0.51	0.06 (−0.39, 0.51)	0.80	0.31 (−0.18, 0.80)	0.22	−0.02 (−0.04, 0.01)	0.21

β: Regression coefficient; CI: confidence interval; HRT: hormonal replacement therapy.

The analysis for epigenetic age acceleration was adjusted for age, BMI and smoking status, and the analysis for genome-wide average DNA methylation was adjusted for age, BMI, smoking status and blood cell composition.

^a^Value are reported as percentage methylation.

Similar results were found from the sensitivity analyses. For categorized BMI, positive associations were found for the Horvath clock: regression coefficient (β) = 1.10, 95% confidence interval (CI) = 0.03 to 2.17, *P* = 0.04 for overweight women, and β = 1.12, 95% CI = 0.08 to 2.17, *P* = 0.04 for obese women. As to the Levine clock, the association for obese women was marginally significant: β = 1.29, 95% CI = −0.01 to 2.58, *P* = 0.05. No association was found for the Hannum clock or genome-wide average DNA methylation. The analyses including the whole sample for pack-years of smoking, number of live births, length of HRT use and length of oral contraceptive use gave similar results (data not shown).

### Replication and meta-analyses

The associated risk factors found above were examined using the Melbourne Collaborative Cohort Study (MCCS) sample (Table 3). At the level of nominal significance (*P* < 0.05), the association between BMI and the Levine clock was replicated using both the MCCS women and men, and the association between pack-years of smoking and the Levine clock was replicated using the MCCS men.

**Table 3:** Association estimates between DNA methylation-based risk factors and conventional risk factors from the MCCS and meta-analysis.

Associations	MCCS women		MCCS men		AMDTSS + MCCS women		AMDTSS + MCCS women + MCCS men	
	β (95% CI)	*P*	β (95% CI)	*P*	β (95% CI)	*P*	β (95% CI)^a^	*P*
Hannum clock								
Age at first live birth	−0.01 (−0.08, 0.06)	0.79			−0.04 (−0.10, 0.01)	0.14		
Ever smoking	0.41 (−0.20, 1.02)	0.19	0.16 (−0.34, 0.65)	0.53	0.11 (−0.37, 0.60)	0.65	0.08 (−0.28, 0.45)	0.66
Horvath clock								
Body mass index	1.51 (−0.41, 3.43)	0.12	1.37 (−0.68, 3.41)	0.19	1.86 (0.38, 3.33)	0.01	1.69 (0.49, 2.89)	5.6E-03
Age at menarche	0.13 (−0.08, 0.33)	0.23			0.18 (0.02, 0.35)	0.03		
Ever smoking	1.03 (0.36, 1.71)	2.8E-03	0.20 (−0.35, 0.74)	0.48	0.77 (0.22, 1.31)	5.8E-03	0.48 (0.10, 0.86)	0.01
Levine clock								
Body mass index	3.03 (0.57, 5.48)	0.02	4.80 (2.27, 7.34)	2.1E-04	3.07 (1.24, 4.90)	1.0E-03	3.66 (2.18, 5.15)	1.3E-06
Pack-years of smoking	0.28 (−0.26, 0.81)	0.31	0.69 (0.33, 1.04)	1.4E-04	0.54 (0.20, 0.88)	2.1E-03	0.61 (0.36, 0.86)	1.2E-06
Alcohol drinking	0.09 (−0.75, 0.93)	0.84	0.75 (−0.17, 1.66)	0.11	0.54 (−0.12, 1.19)	0.11	0.61 (0.08, 1.14)	0.02
Ever smoking	1.22 (0.34, 2.11)	6.9E-03	1.21 (0.54, 1.88)	4.2E-04	0.90 (0.25, 1.58)	0.01	1.06 (0.58, 1.54)	1.4E-05
Genome-wide average DNA methylation^a^								
Number of live births	0.03 (−0.07, 0.12)	0.56			−0.05 (−0.10, 0.004)	0.07		
Age at first live birth	0.001 (−0.01, 0.01)	0.85			0.01 (0.00, 0.01)	0.04		
Ever smoking	−0.06 (−0.14, 0.03)	0.19	−0.04 (−0.09, 0.004)	0.08	−0.04 (−0.09, 0.02)	0.20	−0.04 (−0.07, −0.01)	0.03

β: Regression coefficient; CI: confidence interval.

^a^Values are reported as percentage methylation.

The associations for the Hannum clock were not statistically significant from any of the meta-analyses, and the rest of the associations were found to be nominally significant in at least one of the meta-analyses, with the exception that the association between number of live births and genome-wide average DNA methylation was marginally significant (*P* = 0.07). Smoking status was associated with the greatest number of DNA methylation-based risk factors investigated: the Horvath clock, Levine clock and genome-wide average DNA methylation.

No evidence of heterogeneity by sex was found for any association, except for the association between smoking status and the Horvath clock (*P* = 0.04). Note that this association was also significant in the meta-analysis when including women only.

### Within-twin-pair associations

The associated risk factors found above were examined using within-twin-pair analyses (Table 4). For all twin pairs, there was evidence of a within-twin-pair association between the Horvath clock and BMI. There was no evidence of within-twin-pair association heterogeneity between MZ and DZ pairs (all *P* > 0.05).

**Table 4:** Within-twin-pair association estimates between DNA methylation-based risk factors and conventional risk factors.

Associations^a^	All twin pairs		Monozygotic twin pairs		Dizygotic twin pairs		*P* for heterogeneity
	β (95% CI)	*P*	β (95% CI)	*P*	β (95% CI)	*P*	
Horvath clock							
Body mass index	6.26 (1.65, 10.87)	8.8E-03	5.92 (−0.79, 12.62)	0.09	6.48 (−0.01, 12.97)	0.05	0.87
Age at menarche	0.24 (−0.40, 0.87)	0.47	0.25 (−0.86, 1.36)	0.66	0.23 (−0.60, 1.07)	0.59	0.72
Ever smoking	1.09 (−0.53, 2.72)	0.19	0.89 (−1.25, 3.03)	0.42	1.27 (−1.19, 3.73)	0.32	0.95
Levine clock							
Body mass index	4.70 (−0.76, 10.15)	0.09	4.65 (−3.33, 12.62)	0.26	4.81 (−2.85, 12.47)	0.22	0.84
Pack-years of smoking	0.61 (−0.60, 1.81)	0.33	0.41 (−1.03, 1.84)	0.59	0.87 (−1.47, 3.20)	0.49	0.69
Alcohol drinking	0.50 (−1.50, 2.50)	0.63	0.31 (−2.61, 3.23)	0.83	0.61 (−2.23, 3.45)	0.68	0.92
Ever smoking	1.21 (−0.72, 3.14)	0.22	0.70 (−1.84, 3.25)	0.59	1.63 (−1.27, 4.54)	0.27	0.84
Genome-wide average DNA methylation^b^							
Number of live births	0.01 (−0.13, 0.14)	0.93	0.01 (−0.20, 0.22)	0.94	−0.001 (−0.21, 0.20)	0.99	0.80
Age at first live birth	−0.001 (−0.01, 0.01)	0.96	0.001 (−0.02, 0.02)	0.82	−0.01 (−0.02, 0.01)	0.46	0.53
Ever smoking	−0.05 (−0.14, 0.04)	0.28	0.01 (−0.13, 0.14)	0.93	−0.10 (−0.23, 0.03)	0.14	0.26

^a^The number of pairs included in the analysis for all twin pairs, monozygotic twin pairs and dizygotic twin pairs was 105, 53 and 52 for age at first live birth, 132, 66 and 66 for body mass index, age at menarche, smoking and alcohol drinking, 26, 15 and 11 for pack-years of smoking, and 105, 52 and 52 for number of live births; ^b^Values are reported as percentage methylation.

## Discussion

Using a sample of unaffected women comparable with those in the previous studies^3,7,11,13^ which have reported that DNAm age and genome-wide average DNA methylation are predictive of breast cancer risk, we investigated the associations between these DNA methylation-based risk factors and multiple conventional breast cancer risk factors. We further conducted replication and meta-analyses using independent and homogenous samples, as well as within-twin-pair analyses that control for familial confounding. We found that DNAm age was associated with lifestyle factors (BMI, smoking and alcohol drinking) and hormonal factors (age at menarche), while genome-wide average DNA methylation was associated with hormonal factors (number of live births and age at first live birth) and smoking. We also found evidence that the association of DNAm age with BMI remained after controlling for familial factors shared by twins.

Following our previous finding that the variance in genome-wide average DNA methylation is determined to a large extent by as yet unmeasured environmental factors across the human lifespan, including non-genetic factors shared by relatives^17^, here we found that smoking, number of live births and age at first live birth were associated with this summary measure of DNA methylation across the genome. To the best of our knowledge, our study is the first to report that genome-wide average DNA methylation is associated with these three conventional risk factors.

Similar to previous studies^14,27–30^, we also found DNAm age was positively associated with lifestyle risk factors including BMI, smoking and alcohol drinking. This suggests that some lifestyle factors accelerate biological ageing, consistent with both DNAm age and those lifestyle factors being associated with an increased risk of breast cancer. We found that the Levine clock was associated with more lifestyle factors than the other two clocks. Similar findings have arisen from previous studies; see Table 1 of Horvath *et al*.^31^ Interestingly, the Levine clock was found to be most strongly associated with breast cancer risk^16^. These differences might be due to differences in the development of these clocks; the Horvath and Hannum clocks are trained on chronological age^12,13^, while the Levine clock is trained on the ‘phenotypic age’ which in addition to chronological age also considers nine other health-related factors, so is theoretically more relevant to health^14^.

We found that DNAm age was associated with hormonal risk factors, as has been previously found^26,32^. Binder and colleagues found a negative association between the Horvath clock and age at menarche^32^, which is in the opposite direction of our observed association. One difference between the two studies is that Binder *et al*. studied adolescent girls while we studied older women. Note that the point estimates for the associations of age at menarche and the other two clocks were also positive, providing some more evidence about the direction of association between DNAm age and age at menarche for older women.

Results from the within-twin-pair analyses suggest that our observed association of the Horvath clock with BMI is unlikely to be due to confounding due to familial factors shared by twins, because such potentially confounding effects are cancelled out when using a regression analysis of within-twin-pair differences. In this sense, this association is more likely to be causal. We did not find evidence for genetic confounders, given that the within-twin-pair association was similar for MZ and DZ twin pairs. The null results for the other within-twin-pair associations do not necessarily imply that the associations observed in Tables 2 and 3 are due to familial confounding. Note that, the confidence interval of the within-twin-pair association in Table 4 contained the point estimate of the corresponding association in Tables 2 and 3, suggesting that the associations tend to be alike, and the lack of statistical significance in the within-twin-pair analyses is possibly a result of insufficient sample size.

Our findings imply that DNA methylation-based risk factors potentially interplay with their associated conventional risk factors in modifying breast cancer risk. Such interplay may be the due to the DNA methylation-based risk factors mediating the associations between conventional risk factors and cancer risk, or other mechanisms. Studies with appropriate data, design and analytic methods are required to further investigate the interrelationships between DNA methylation-based risk factors, conventional risk factors and breast cancer risk. Findings from such studies could provide novel insights on the aetiology, early detection and prevention of breast cancer.

The strengths of our study include: (1) using samples comparable with those used in studies that reported the DNA methylation-based risk factors, (2) investigating a comprehensive list of conventional breast cancer risk factors, which notably includes family history and mammographic density, and (3) using a sample for discovery and homogenous samples for replication analyses, as well as conducting a meta-analysis to take advantage of the increased sample size. One potential limitation is that no multiple testing adjustment was performed; our results must of course be interpreted with this in mind.

In conclusion, our study found evidence that the lifestyle risk factors BMI, smoking and alcohol drinking, and the hormonal breast cancer risk factors age at menarche, age at first live birth and number of liver births, are associated with the DNA methylation-based biological age and global DNA methylation. We also found evidence that the observed associations are unlikely to be due to familial confounding but are likely causal. DNA methylation-based risk factors could interplay with conventional risk factors to modify breast cancer risk. Such interplay requires further investigation.

## Methods

### Study sample for discovery

The sample was participants from the Australian Mammographic Density Twins and Sisters Study (AMDTSS)^33^, which was approved by Human Research Ethics Committee at the University of Melbourne in accordance with the Declaration of Helsinki. All participants provided written informed consent. The analytical sample included 479 women from 130 families: 66 monozygotic twin (MZ) pairs, 66 dizygotic twin (DZ) pairs and 215 sisters of twins^34^. They were aged from 40–78 years and had a mean (standard deviation [SD]) age of 55.7 (8.0) years. All women were healthy and none had been diagnosed with breast cancer when recruited.

### DNAm age and genome-wide average DNA methylation

Methylation of DNA extracted from dried peripheral blood spots was measured using the HM450 assay. Data were normalized using Illumina’s reference factor-based normalization methods and subset-quantile within array normalization^35^ for type I and II probe bias correction, and an empirical Bayes batch effects removal method, *ComBat*^36^, was applied to minimise technical variation across physical batches; see Li *et al*.^34^ for more details.

DNAm age was calculated based on each of the Hannum, Horvath and Levine clocks, using the online calculator (https://dnamage.genetics.ucla.edu). As done previously^16^, we investigated epigenetic age acceleration, calculated as the residuals from regressing DNAm age on chronological age and blood cell composition. Therefore, epigenetic age acceleration was independent of chronological age and blood cell composition. Blood cell composition (CD8 + T-cells, CD4 + T-cells, natural killer cells, B-cells, monocytes and granulocytes) was estimated using the Houseman method^37^ implemented in the *minfi* package and Reinius *et al*.^38^ as reference. Genome-wide average DNA methylation was calculated as the average methylation beta-value across all autosomal probes that passed quality control.

### Conventional breast cancer risk factors

We studied multiple conventional breast cancer risk factors, including lifestyle factors, hormonal factors, family history and mammographic density (Table 1). All risk factors except mammographic density were collected via telephone-administered questionnaire survey. Lifestyle factors included body mass index (BMI), smoking status, smoking intensity measured as pack-years and alcohol drinking. Hormonal factors included age at menarche, parity, number of live births, age at first live birth, oral contraceptive use, length of oral contraceptive use in years, menopausal status, age at menopause, hormonal replacement therapy (HRT) use and duration of HRT use in years. Family history was defined as having at least one first-degree relative diagnosed with breast cancer. Mammographic density was measured using the computer-assisted thresholding technique, CUMULUS (Imaging Research Program, Sunnybrook Health Sciences Centre, University of Toronto, Toronto, Canada), at the conventional brightness threshold. We studied three measures: dense area, non-dense area and percentage dense area. Details of the measurement can be found in Odefrey *et al*.^33^ 435 women had mammographic density data available.

### Statistical analysis

Correlations between DNA methylation-based risk factors and chronological age were assessed using Pearson’s correlation coefficient. We investigated the association of each DNA methylation-based risk factor (the three epigenetic age acceleration measures and genome-wide average DNA methylation) with the conventional risk factors separately using a linear regression model, in which the DNA methylation-based risk factor was the dependent variable and the conventional risk factor was the independent variable. To account for the relatedness between family members, the regression model was fitted using the Generalised Estimating Equations method with family as cluster. The model was adjusted for age, BMI, smoking status and blood cell composition, with the exception that blood cell composition was dropped from the model investigating epigenetic age acceleration, since epigenetic age acceleration is independent of cell composition by construction. A *P*-value of 0.05 was used to define nominal statistical significance. All statistical tests were two-sided.

Mammographic dense, non-dense and percentage dense areas were power-transformed with powers of 0.25, 0.25, 0.4, respectively, and the other right-skewed continuous risk factors (BMI, pack-years of smoking, number of live births, and durations of HRT and oral contraceptive use) were log transformed, to have an approximately normal distribution. The analyses for pack-years of smoking, age at menopause, number of live births, age at first live birth, length of HRT use and length of oral contraceptive use, were restricted to participants who were ever smokers, post-menopausal, parous, ever HRT users and ever oral contraceptive users, as appropriate.

### Sensitivity analysis

BMI was analysed as a categorical variable: normal (BMI < 25; 217 women; used as the reference group), overweight (25 ≤ BMI < 30; 146 women) and obese (BMI ≥ 30; 116 women). The analyses for pack-years of smoking, number of live births, length of HRT use and length of oral contraceptive use included non-smokers, nulliparous women, HRT non-users and oral contraceptive non-users as well, for whom the corresponding variable was treated as zero and added by one in log transformation.

### Replication and meta-analysis

For the associations with a nominal *P* < 0.05, we performed replication analyses using the data of participants (1,350 women, 2,004 men) from the Melbourne Collaborative Cohort Study (MCCS)^39^. The participants were the controls from case-control studies of several cancers nested in the MCCS. Same as those in the AMDTSS, DNA samples were extracted from peripheral blood, DNA methylation data were measured using the HM450 assay and normalized using Illumina’s reference factor-based normalization methods and subset-quantile within array normalization; see Dugue *et al*.^23,27^ for more details.

The association was investigated using the same linear regression model. Additional to the covariates used in the AMDTSS analysis, country of birth (Australia/New Zealand, United Kingdom/Malta, Italy, or Greece) and sample type (dried blood spot, peripheral blood mononuclear cells, or buffy coat) were adjusted for, all fitted as fixed-effects. Batch effects were minimized by fitting study and the plate on which the sample was processed as random effects. We also investigated the associations between smoking status and DNA methylation-based risk factors. The analysis was stratified by sex. The heterogeneity in the association by sex was investigated by fitting an interaction term of the risk factor with sex in the regression model for the whole sample.

Taking advantage of the homogeneity between the two samples^40^, results from the two studies were pooled via a fixed-effect meta-analysis, using the generic inverse variance method. Two meta-analyses were performed: (1) AMDTSS sample and MCCS women, and (2) AMDTSS sample, MCCS women and MCCS men.

### Within-twin-pair analysis

Taking advantage of our sample including twin pairs, we performed within-twin-pair analyses to investigate the associations after controlling for the confounding effects of familial factors (both known and unknown) shared by twins. Within-twin-pair differences in the DNA methylation-based risk factors, conventional risk factors and covariates were calculated, and used to estimate their within-twin-pair associations using an ordinary linear regression model without an intercept^41^. The within-twin-pair analyses were performed separately for all twin pairs, MZ pairs and DZ pairs. We investigated the heterogeneity in the within-twin-pair association by zygosity by fitting an interaction term of the risk factor with zygosity in the regression model for all twin pairs.

## Data Availability

The DNA methylation dataset of the AMDTSS can be accessed on Gene Expression Omnibus with the number GSE100227.

## References

[1] Esteller M (2008). Epigenetics in cancer. The New England journal of medicine.

[2] Petronis A (2010). Epigenetics as a unifying principle in the aetiology of complex traits and diseases. Nature.

[3] Wong EM (2011). Constitutional methylation of the BRCA1 promoter is specifically associated with BRCA1 mutation-associated pathology in early-onset breast cancer. Cancer prevention research (Philadelphia, Pa.).

[4] Iwamoto T, Yamamoto N, Taguchi T, Tamaki Y, Noguchi S (2011). BRCA1 promoter methylation in peripheral blood cells is associated with increased risk of breast cancer with BRCA1 promoter methylation. Breast cancer research and treatment.

[5] Brennan K (2012). Intragenic ATM methylation in peripheral blood DNA as a biomarker of breast cancer risk. Cancer research.

[6] Xu Z (2013). Epigenome-wide association study of breast cancer using prospectively collected sister study samples. Journal of the National Cancer Institute.

[7] Heyn H (2013). DNA methylation profiling in breast cancer discordant identical twins identifies DOK7 as novel epigenetic biomarker. Carcinogenesis.

[8] Severi G (2014). Epigenome-wide methylation in DNA from peripheral blood as a marker of risk for breast cancer. Breast cancer research and treatment.

[9] van Veldhoven K (2015). Epigenome-wide association study reveals decreased average methylation levels years before breast cancer diagnosis. Clinical epigenetics.

[10] Xu, Z., Sandler, D. P. & Taylor, J. A. Blood DNA methylation and breast cancer: A prospective case-cohort analysis in the Sister Study. *Journal of the National Cancer Institute*, 10.1093/jnci/djz065 (2019). 10.1093/jnci/djz065 PMC7489106 30989176

[11] Dugué, P.-A., Li, S., Hopper, J. L. & Milne, R. L. In *Epigenetics in Human Disease* Vol. 6 (ed Trygve O. Tollefsbol) 39–64 (Academic Press, 2018).

[12] Hannum G (2013). Genome-wide methylation profiles reveal quantitative views of human aging rates. Molecular cell.

[13] Horvath S (2013). DNA methylation age of human tissues and cell types. Genome Biol.

[14] Levine ME (2018). An epigenetic biomarker of aging for lifespan and healthspan. Aging.

[15] Ambatipudi S (2017). DNA methylome analysis identifies accelerated epigenetic ageing associated with postmenopausal breast cancer susceptibility. European journal of cancer (Oxford, England: 1990).

[16] Kresovich, J. K. *et al*. Methylation-based biological age and breast cancer risk. *Journal of the National Cancer Institute*, 10.1093/jnci/djz020 (2019). 10.1093/jnci/djz020 PMC6792078 30794318

[17] Li S (2018). Genome-wide average DNA methylation is determined in utero. International journal of epidemiology.

[18] Vryer R, Saffery R (2017). What’s in a name? Context-dependent significance of ‘global’ methylation measures in human health and disease. Clinical epigenetics.

[19] Deroo LA (2014). Global DNA methylation and one-carbon metabolism gene polymorphisms and the risk of breast cancer in the Sister Study. Carcinogenesis.

[20] Kuchiba A (2014). Global methylation levels in peripheral blood leukocyte DNA by LUMA and breast cancer: a case-control study in Japanese women. British journal of cancer.

[21] Bodelon C (2019). Blood DNA methylation and breast cancer risk: a meta-analysis of four prospective cohort studies. Breast cancer research: BCR.

[22] Wong Doo N (2016). Global measures of peripheral blood-derived DNA methylation as a risk factor in the development of mature B-cell neoplasms. Epigenomics.

[23] Dugue PA (2016). Genome-wide measures of DNA methylation in peripheral blood and the risk of urothelial cell carcinoma: a prospective nested case-control study. British journal of cancer.

[24] FitzGerald LM (2017). Genome-Wide Measures of Peripheral Blood Dna Methylation and Prostate Cancer Risk in a Prospective Nested Case-Control Study. The Prostate.

[25] Christensen, B. Your DNA may appear older than you think. *Journal of the National Cancer Institute*, 10.1093/jnci/djz021 (2019). 10.1093/jnci/djz021 PMC6792063 30794313

[26] Levine ME (2016). Menopause accelerates biological aging. Proceedings of the National Academy of Sciences of the United States of America.

[27] Dugue PA (2018). Association of DNA Methylation-Based Biological Age With Health Risk Factors and Overall and Cause-Specific Mortality. American journal of epidemiology.

[28] Quach A (2017). Epigenetic clock analysis of diet, exercise, education, and lifestyle factors. Aging.

[29] Fiorito G (2017). Social adversity and epigenetic aging: a multi-cohort study on socioeconomic differences in peripheral blood DNA methylation. Sci Rep.

[30] Nevalainen T (2017). Obesity accelerates epigenetic aging in middle-aged but not in elderly individuals. Clinical epigenetics.

[31] Horvath S, Raj K (2018). DNA methylation-based biomarkers and the epigenetic clock theory of ageing. Nature reviews. Genetics.

[32] Binder AM (2018). Faster ticking rate of the epigenetic clock is associated with faster pubertal development in girls. Epigenetics.

[33] Odefrey F (2010). Common genetic variants associated with breast cancer and mammographic density measures that predict disease. Cancer research.

[34] Li S (2015). Genetic and Environmental Causes of Variation in the Difference Between Biological Age Based on DNA Methylation and Chronological Age for Middle-Aged Women. Twin research and human genetics: the official journal of the International Society for Twin Studies.

[35] Maksimovic J, Gordon L, Oshlack A (2012). SWAN: Subset-quantile within array normalization for illumina infinium HumanMethylation450 BeadChips. Genome Biol.

[36] Johnson WE, Li C, Rabinovic A (2007). Adjusting batch effects in microarray expression data using empirical Bayes methods. Biostatistics.

[37] Houseman EA (2012). DNA methylation arrays as surrogate measures of cell mixture distribution. BMC bioinformatics.

[38] Reinius LE (2012). Differential DNA methylation in purified human blood cells: implications for cell lineage and studies on disease susceptibility. PLoS One.

[39] Milne RL (2017). Cohort Profile: The Melbourne Collaborative Cohort Study (Health 2020). International journal of epidemiology.

[40] Li S (2019). Genome-wide association study of peripheral blood DNA methylation and conventional mammographic density measures. Int J Cancer.

[41] Hopper JL, Seeman E (1994). The bone density of female twins discordant for tobacco use. The New England journal of medicine.

